# Dual-specificity MAP kinase phosphatases (MKPs): Shaping the outcome of MAP kinase signalling

**DOI:** 10.1111/j.1742-4658.2012.08716.x

**Published:** 2013-01

**Authors:** Christopher J Caunt, Stephen M Keyse

**Affiliations:** 1 Department of Biology and Biochemistry, University of BathUK; 2 CR-UK Stress Response Laboratory, Medical Research Institute, Ninewells Hospital & Medical SchoolDundee, UK

**Keywords:** dual specificity protein phosphatase, DUSP, ERK, JNK mitogen activated protein kinase, MAPK, MAPK localisation, MAPK phosphatase, MKP, p38

## Abstract

Dual-specificity MAP kinase phosphatases (MKPs) provide a complex negative regulatory network that acts to shape the duration, magnitude and spatiotemporal profile of MAP kinase activities in response to both physiological and pathological stimuli. Individual MKPs may exhibit either exquisite specificity towards a single mitogen-activated protein kinase (MAPK) isoform or be able to regulate multiple MAPK pathways in a single cell or tissue. They can act as negative feedback regulators of MAPK activity, but can also provide mechanisms of crosstalk between distinct MAPK pathways and between MAPK signalling and other intracellular signalling modules. In this review, we explore the current state of knowledge with respect to the regulation of MKP expression levels and activities, the mechanisms by which individual MKPs recognize and interact with different MAPK isoforms and their role in the spatiotemporal regulation of MAPK signalling.

## Introduction

Mitogen-activated protein kinases (MAPKs) are components of highly conserved signal transduction pathways, which act in a concerted manner to determine both physiological and pathological responses to a wide variety of extracellular and intracellular stimuli [1,2]. These proline-directed Ser/Thr kinases regulate processes such as gene expression, protein translation, protein stability, protein localization and enzyme activity, thus affecting diverse cellular endpoints including cell proliferation, differentiation, cell survival and cell death [3,4]. Given this range of functions, it is no surprise that MAPKs play key roles in a wide range of physiological processes, including embryogenesis, innate and adaptive immunity, metabolic homeostasis, cardiac function and neuronal plasticity, or that abnormalities in MAPK signalling are associated with human diseases, including obesity/diabetes, rheumatoid arthritis, neurodegenerative disorders and cancer [5–8].

MAPK pathways all share a common architecture of a three-tiered kinase cascade comprised of a MAPK kinase kinase (MKKK or MEKK), a MAPK kinase (MKK or MEK) and the MAPK itself where activation results from the sequential phosphorylation and activation of each component kinase in turn [9]. The MAPKs are unusual in requiring dual phosphorylation of both a Thr and a Tyr residue within the signature motif T–X–Y in the activation loop of the kinase for activity, and thus the MKK acts as a dual-specificity (Thr/Tyr) protein kinase. There are four major MAPK pathways in mammalian cells. These are the classical Ras/MAPK pathway containing extracellular signal-regulated kinases (ERKs) 1 and 2 (also known as p44 and p42 MAPKs or MAPK1 and MAPK2, respectively), the p38 family of MAPKs comprising p38α, p38β, p38δ and p38γ, the c-jun N-terminal kinases (or JNKs) 1, 2 and 3 and the ERK5 pathway [10]. In addition, there are MAPKs such as ERK7/8 and ‘atypical’ MAPKs typified by ERK3 and ERK4, the functions of which are less well understood [11].

One highly conserved property of MAPK pathways is that the duration and magnitude of MAPK activation plays a major role in determining the biological outcome of signalling [12]. Pathway output thus reflects a balance between the activity of upstream pathway components and various negative regulatory mechanisms. Although the latter can operate at multiple points in the pathway, including at the level of cell-surface receptors, it is now clear that the MAPK itself is subject to negative regulation by specific protein phosphatases acting in direct opposition to the MKK to attenuate MAPK signalling. Because MAPKs require phosphorylation of both the threonine and tyrosine residues within the activation loop, dephosphorylation of either residue can inactivate the kinase. This function can be performed by type 1/2 Ser/Thr phosphatases, protein tyrosine phosphatases or by dual-specificity (Thr/Tyr) protein phosphatases, and studies in a wide variety of model organisms have shown that all three classes of protein phosphatases may be involved [13]. However, by far the largest group of protein phosphatases dedicated to the specific regulation of MAPK activity in mammalian cells and tissues are the dual-specificity MAPK phosphatases (MKPs or DUSPs).

## The dual-specificity MAPK phosphatase gene family

**Fig. 1:**
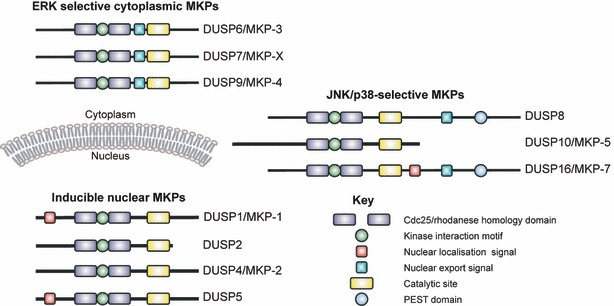
Classification, localization and domain structure of the MKPs. The three subclasses of MKPs are grouped according to localization, substrate specificity and sequence similarity. The JNK/p38 phosphatases are evenly distributed between the nucleus and cytoplasm and so are not represented on either side of the nuclear envelope here. Positions of the major features of MKPs within the primary amino acid sequences of MKPs are represented as defined in the key.

MKPs represent a distinct subfamily within a larger group of dual-specificity protein phosphatases. The latter include the so-called ‘atypical’ DUSPs, which have a rather ill-defined and complex relationship with MAPK activation, being reported to mediate both positive and negative regulation of these pathways [14]. There are 10 catalytically active MKPs in mammalian cells, and these all share a common structure consisting of an N-terminal noncatalytic domain and a C-terminal catalytic domain, the latter of which contains the PTPase consensus active site sequence [15]. It is now clear that the N-terminal domain has regulatory functions. It contains a modular docking site, which mediates the ability of the phosphatase to recognize and regulate specific MAPK isoforms, and also sequences that determine the subcellular localization of the phosphatase [16]. Based on sequence homology, subcellular localization and substrate specificity, the 10 MKPs can be subdivided into three subfamilies (Fig. 1). The first of these includes DUSP1/MKP-1, DUSP2 (PAC1), DUSP4/MKP-2 and DUSP5, all of which are mitogen- and stress-inducible nuclear MKPs. The second group comprises DUSP6/MKP-3, DUSP7/MKP-X and DUSP9/MKP-4, which are cytoplasmic ERK-specific MKPs. The final group comprising DUSP8 (M3/6), DUSP10/MKP-5 and DUSP16/MKP-7 are JNK/p38-specific phosphatases, which are found in both the cell nucleus and cytoplasm [17,18].

## Substrate specificity and catalytic mechanism

Following the characterization of the cytoplasmic phosphatase DUSP6/MKP-3 (also known as Pyst1 or rVH-6), it was quickly realized that this enzyme could bind specifically to the classical ERK1 and ERK2 MAPKs *in vitro*, and that this binding defined its ability to specifically dephosphorylate and inactivate these MAPKs *in vivo* [19,20]. This contrasted with the activity of either DUSP8 (M3/6), which does not inactivate ERK, but is specific for p38 and JNK [20], or the inducible nuclear phosphatase DUSP1/MKP-1, which can bind and dephosphorylate all three major classes of MAPK (ERK, p38 and JNK) both *in vitro* and *in vivo* [21,22]. Subsequent work identified a kinase interaction motif (KIM) within the N-terminus of the MKPs, which was responsible for MAPK binding, and also defined cognate motifs in the MAPKs, which were responsible for high-affinity interactions with the KIM [23,24]. The core of the KIM in the MKPs is characterized by a cluster of two to three positively charged Arg residues. These, together with an additional motif of positively charged residues flanked by hydrophobic amino acids (Leu, Ile or Val), comprise a modular binding domain in which variations in the numbers and positions of the positively charged and hydrophobic residues are thought to contribute to the specificity of MAPK binding [25]. These motifs engage with a ‘common docking’ (CD) site comprised of negatively charged Asp residues and additional sequence determinants, including the ED motif and an extended docking groove on the MAPK [24,26,27]. The primary importance of the positively charged residues within the KIM of DUSP6/MKP-3 in mediating its interaction with ERK2 was confirmed by the crystal structure of a KIM peptide bound to ERK2. This revealed that the vast majority of the side-chain contacts in this complex are provided by Arg65 within the DUSP6/MKP-3 KIM and Asp319 within the CD of ERK2 [28]. This confirmed the results of previous experiments in which mutation of either Arg65 in DUSP6/MKP-3 or Asp319 in ERK2 abrogated the interaction between the two proteins [23,29].

More recently, the crystal structure of a complex between the MAPK binding domain of DUSP10/MKP-5 and its substrate p38α MAPK has been obtained. Surprisingly, this has revealed a novel interaction mode for DUSP10/MKP-5 in that, although the positively charged arginine residues (Arg203 and 204) of the KIM are still essential for binding to the CD domain of p38α, the orientation and binding mechanism is completely different [30]. First, the putative hydrophobic motif in the DUSP10/MKP-5 KIM does not play a significant role in p38α binding. Second, the KIM peptide binds to the docking surface in p38α in the opposite polypeptide direction when compared with DUSP6/MKP-3, binding p38α such that two distinct helical regions within the MAPK-binding domain of DUSP10/MKP-5 engage the p38α docking site. This difference in binding mode is mirrored in distinct structural features of the two MAPK-binding domains. Whereas the KIM peptide in DUSP6/MKP-3 is located in a flexible region lying between a beta sheet (β3) and an alpha helix (α3), the equivalent sequence in DUSP10/MKP-5 is located in an alpha helix (α3′) and a following loop and lies on the opposite face of the central β sheet. Based on structural similarity between the MAPK-binding domain of DUSP10/MKP-5 and DUSP16/MKP-7, the authors suggest that this mechanism may be a conserved feature of the p38 and JNK-specific phosphatases DUSP10/MKP-5, DUSP8 (M3/6) and DUSP16/MKP-7 and may underpin their ability to interact specifically with these kinases rather than with ERK [30]. This is certainly a possibility, but it should be remembered that in the nuclear phosphatase DUSP1/MKP-1, which can interact with ERK, JNK and p38, the KIM is only required for interaction with ERK and p38. A DUSP1/MKP-1 mutant in which Arg53–55 have been substituted by Ala fails to bind to either ERK2 or p38, but can interact with and inactivate JNK both *in vitro* and *in vivo* just as efficiently as the wild-type protein [22]. The same is true for the related enzyme DUSP4/MKP-2 [31]. These observations suggest that there must be even more complexity to the differential binding modes of the MKPs than has been uncovered thus far.

A further twist to the specific interactions between MKPs and their cognate MAPKs came with the discovery that the binding of DUSP6/MKP-3 to ERK2 is associated with catalytic activation of the bound phosphatase *in vitro* [32]. Subsequent biochemical and structural studies demonstrated that activation resulted from conformational changes within the catalytic domain of the protein and, primarily, the movement of a loop containing a conserved Asp residue, which acts as a general acid during catalysis such that this residue is positioned optimally to perform its function [33–35]. It was quickly realized that binding and catalytic activation were largely predictive of substrate selectivity for a number of MKPs. These include DUSP1/MKP-1, DUSP4/MKP-2 and DUSP2 (PAC1) [22,31,36]. However, the ability to undergo catalytic activation is not a universal property of MKPs. For example, the ERK-specific nuclear phosphatase DUSP5 binds tightly to ERK2, but this binding does not increase the basal activity of the enzyme [37], and the JNK- and p38-specific phosphatase DUSP10/MKP-5 is not activated on binding to these MAPKs [38]. The crystal structures of the catalytic domains of these enzymes provide an explanation for this because, unlike in DUSP6/MKP-3, the general acid-containing loop in both DUSP5 and DUSP10/MKP-5 is already in the optimal position to participate in catalysis [39,40]. To date, it is completely unclear why some MKPs undergo catalytic activation whereas others do not.

## Are there non-MAPK substrates for the dual-specificity MKPs?

Thus far, we know that MKPs are highly specific in their ability to recognize and bind to MAPK substrates. For example, despite the fact that DUSP1/MKP-1 is capable of binding to ERK, p38α and JNK, and might be considered promiscuous in its ability to recognize different MAPK substrates, it is totally unable to recognize and inactivate either p38δ or p38γ, both of which are almost 65% identical to p38α at the amino acid sequence level [22]. This, coupled with the fact that several of the MKPs including DUSP1/MKP-1 undergo catalytic activation on binding to cognate MAPK substrates and have very low basal activities in their absence, must constrain their ability to dephosphorylate potential non-MAPK substrates. For example, based on anti-sense mRNA knockdown experiments, it was suggested that DUSP1/MKP-1 could interact with and dephosphorylate the signal transducers and activators of transcription (STAT) 1 protein, thus regulating transcriptional responses to interferon γ [41]. However, subsequent *in vitro* assays demonstrated that DUSP1/MKP-1 failed to bind to recombinant STAT1, nor was STAT1 able to stimulate the catalytic activity of DUSP1/MKP-1 towards the chromogenic substrate *para*-nitrophenyl phosphate. Finally, while overexpression of DUSP1/MKP-1 efficiently suppressed MAPK-dependent transcription, it had no effect at all on the activity of an interferon-γ-dependent transcriptional reporter, nor did it result in dephosphorylation of STAT1 in cells stimulated with interferon γ [22]. Overall, these results demonstrate that STAT1 is not a DUSP1/MKP-1 substrate and that this phosphatase plays no role in the regulation of STAT1-dependent transcriptional regulation in response to interferon γ. Subsequent work identified TC45, the nuclear isoform of the T-cell protein tyrosine phosphatase, as the *bona fide* activity responsible for STAT1 dephosphorylation and inactivation [42].

More recently, DUSP1/MKP-1 has been proposed to act as a phosphatase towards phosphorylated histone H3. Specifically, it was found that the dephosphorylation of histone H3 (phospho-serine 10) correlated exactly with the kinetics of DUSP1/MKP-1 protein induction in response to thrombin and vascular endothelial growth factor. Both *in vitro* phosphatase assays and the results of siRNA-mediated knockdown suggested that DUSP1/MKP-1 was the responsible activity [43]. We examined the kinetics of histone H3 (phospho-serine 10) phosphorylation and dephosphorylation in response to mitogen stimulation in wild-type mouse embryonic fibroblasts and compared this with mouse embryonic fibroblasts derived from the DUSP1/MKP-1^−/−^ knockout mouse in which *DUSP1* has been deleted by homologous recombination [44]. In agreement with the previous publication, we found that histone H3 dephosphorylation correlated exactly with the appearance of the DUSP1/MKP-1 protein. However, the kinetics of dephosphorylation were identical in cells lacking DUSP1/MKP-1, indicating that this phosphatase is unlikely to be the activity responsible.

The ERK-specific cytoplasmic phosphatase DUSP6/MKP-3 has been implicated in the positive regulation of gluconeogenic gene expression and is upregulated in the livers of diet-induced obese mice [45]. It is difficult to reconcile these results solely on the basis of a change in ERK activity, and a recent study has suggested that DUSP6/MKP-3 may intervene more directly in this pathway by interacting with and dephosphorylating the transcription factor forkhead box O1 (FOXO1). DUSP6/MKP-3 mediated dephosphorylation of Ser256 in FOXO1 is proposed to promote FOXO1 nuclear import, thus increasing its transcriptional activity [46]. The main evidence implicating DUSP6/MKP-3 in the regulation of FOXO1 was its coimmunoprecipitation with FOXO1 and its ability to dephosphorylate FOXO1 when coexpressed in cells. Although more extensive work is required to test this hypothesis definitively, we expressed recombinant FOXO1 and assessed its ability to catalytically activate DUSP6/MKP-3 *in vitro*. Whereas recombinant ERK2 increased the activity of DUSP6/MKP-3 towards *para*-nitrophenyl phosphate up to 30-fold, FOXO1 did not (Stephen Keyse, unpublished observations). The availability of mice lacking DUSP6/MKP-3 should enable more definitive experiments to further explore the relationship between DUSP6/MKP-3, FOXO1 signalling and the expression of gluconeogenic genes.

Although the ability of MAPKs to bind to and catalytically activate MKPs would appear to impose severe constraints on the ability of these enzymes to directly dephosphorylate non-MAPK substrates, it is formally possible that they may do this while in complex with an (inactive) MAPK. This mechanism has been proposed to account for the ability of the ERK-specific MKP DUSP6/MKP-3 to dephosphorylate p38α MAPK [47]. It is postulated that DUSP6/MKP-3, ERK2 and p38α form a ternary complex in which the phosphatase activity of DUSP6/MKP-3 towards p38α is allosterically regulated by ERK2-DUSP6/MKP-3 interaction. Despite the fact that DUSP9/MKP-4, which is closely related to DUSP6/MKP-3, binds to p38 MAPK using the same conserved Arg residues within the N-terminal KIM motif, it is proposed that a distinct site on p38α that is responsible for productive binding to the catalytic domain of DUSP6/MKP-3 is exposed only when p38α becomes phosphorylated. Again, cells from DUSP6/MKP-3 knockout mice should prove useful in determining the effects of gene loss on p38-dependent endpoints such as activation of MAPKAP kinase-2, particularly under conditions where both ERK and p38 kinases are activated. Finally, it should be noted that certain MKPs such as DUSP5, DUSP8 (M3/6), DUSP10/MKP-5 and DUSP16/MKP-7 do not undergo catalytic activation. Furthermore, DUSP8 (M3/6) and DUSP16/MKP-7 can bind to scaffolding proteins associated with the JNK MAPK pathway making it possible that they are brought into close proximity to non-MAPK components and substrates within these complexes [48,49].

## Subcellular localization of the MKPs

**Fig. 2:**
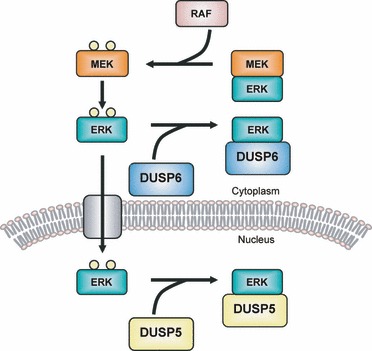
Regulation of ERK distribution by MEK, DUSP5 and DUSP6/MKP-3. The figure illustrates how MEK, DUSP5 and DUSP6/MKP-3 cooperate to regulate ERK responses in the nucleus and cytoplasm, respectively. The cytosolic kinase, MEK is the only known MKK for ERK and anchors ERK in the cytoplasm under basal conditions. Activation of MEK by the RAF MAPK kinase kinase causes phosphorylation of ERK and dissociation of MEK. This commonly causes nuclear accumulation of ERK unless cytoplasmic anchors or scaffolds of ERK are present at sufficient concentration. The NES of DUSP6/MKP-3 and high affinity for ERK binding, irrespective of phosphorylation state enables competition with MEK and other ERK partners and substrates in the cytoplasm, causing sequestration of dephosphorylated ERK in this compartment. DUSP5 is biochemically similar to DUSP6 in its high selectivity for ERK substrates, but is nuclear targeted because of its NLS, and performs an analogous role by sequestering dephosphorylated ERK in the nucleus.

The most striking examples of differential subcellular localization of MKPs are provided by the ERK-specific phosphatases DUSP6/MKP-3 and DUSP5, which are localized in the cytoplasm and nuclear compartments, respectively (Fig. 2). In the case of DUSP6/MKP-3, treatment of cells with leptomycin B, a potent and specific inhibitor of chromosome region maintenance (CRM)-1-dependent nuclear export, causes complete relocalization of the protein to the cell nucleus and led to the identification of a canonical leucine-rich nuclear export signal (NES) within the N-terminal noncatalytic domain of the protein [50]. This NES is conserved in the related proteins DUSP7/MKP-X and DUSP9/MKP-4, indicating a common mechanism by which this subfamily of MKPs is preferentially partitioned into the cytoplasmic compartment. However, the presence of the NES also indicates that these proteins are capable of nuclear–cytoplasmic shuttling.

In the case of DUSP5, again the noncatalytic N-terminus of the protein is responsible for the nuclear targeting of the protein, and a series of deletion and mutation experiments identified a short sequence lying N-terminal to the conserved arginine residues of the KIM, which appears to function as a noncanonical nuclear localization signal (NLS) [37]. A sequence in the same region of the N-terminal noncatalytic domain of the DUSP1/MKP-1 was independently identified as mediating the nuclear localization of that protein [51]. Furthermore, this sequence is not found in cytoplasmic MKPs, indicating that it is conserved only in the nuclear MKPs. Although the N-terminal noncatalytic domains of several MKPs appear to contain either NLS or NES, there are examples where this is not the case. The p38- and JNK-specific phosphatase DUSP16/MKP-7 is unusual in containing a unique C-terminal extension, and a series of experiments identified both a functional leucine-rich NES and a canonical Lys/Arg-rich NLS within this C-terminal domain [52]. Interestingly, despite the location of the NLS in DUSP16/MKP-7, deletion of the N-terminal noncatalytic domain of the protein interfered with its nuclear translocation in the presence of leptomycin B, indicating that this region of the protein plays an important role in determining subcellular localization.

## Spatiotemporal regulation of MAPK signalling by MKPs

**Fig. 3:**
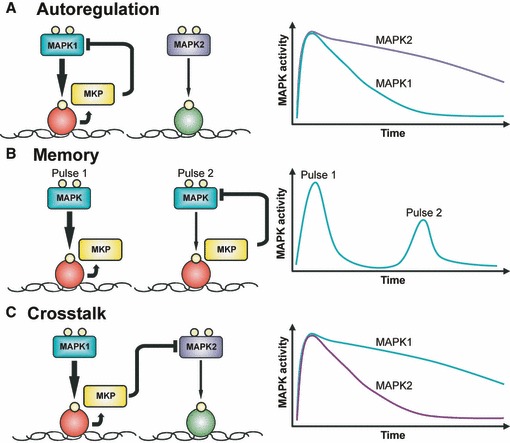
MKPs as temporal regulators of MAPK signalling. The signalling diagrams on the left show how MKPs mediate dynamic effects on MAPK substrates, and the graphs on the right illustrate hypothetical scenarios of how temporal changes in MAPK activity may result from such regulation. MAPK1 and MAPK2 represent notional kinases in these pictures responding to prolonged, slowly desensitizing upstream signals. (A) MKPs can act in autoregulatory loops in response to sustained signals and act to desensitize the MAPK that caused their expression. This serves to attenuate MAPK activity and make it more transient in the face of prolonged upstream activation. (B) MKPs are often induced by transient MAPK signals that are desensitized by mechanisms too rapid to involve MKPs. Thus, the MKP expression may alter the response to subsequent pulses of signal or to other stimuli acting through target MAPKs, enabling the cell to retain a transient ‘memory’ that influences the dynamics of subsequent signalling events. (C) MKPs induced by MAPK activity may have substrate preference for other MAPK isoforms, causing decreases in signal flux through parallel pathways in crosstalk mechanisms. Several examples of each of these scenarios have been defined experimentally, and some of the clearest are highlighted in the main text to illustrate these differential regulatory modes.

As we begin to understand the different biochemical features of MKPs that regulate their expression, localization and catalytic activity, the challenge is to elucidate the precise biological functions that these properties confer. There is apparent redundancy in the negative regulation of MAPK signalling. For example, at least 13 distinct phosphatases can directly dephosphorylate activated ERK alone [16,53]. ERK is dephosphorylated extremely rapidly in certain cell lines following growth factor stimulation or MKK inhibition [54,55], indicating that constitutive Ser/Thr phosphatase activity (mediated by enzymes such as PP1 and PP2A) that act at multiple levels in MAPK cascades [56], coupled with tight control of signal flux through MAPKKK and MKK [57], are major mechanisms for controlling the intensity of MAPK output. By contrast, the dynamic control of expression level, substrate selectivity and subcellular compartmentalization shown by MKPs places them as modulators of sustained MAPK signalling by regulating targeting and crosstalk, rather than simple ‘off’ switches (Figs 2 and 3). As discussed above, characteristic properties of the MKPs make them ideally suited to this role, and are discussed in more depth below.

In contrast to most other phosphatases, there is no requirement for the MAPK substrate to be phosphorylated in order to be recognized and bound by an MKP. For example, DUSP6/MKP-3 association with dual phosphorylated (pp)ERK2 has a *K*_d_ of ∼ 30 nm, but also associates with ERK2 in the dephosphorylated form, albeit with reduced affinity (the *K*_d_ for DUSP6/MKP3 binding to dephosphorylated ERK2 is ∼ 190 nm) [35,58,59]. These dissociation constants are similar to those observed between ERK2 and common substrates assessed using surface plasmon resonance assays. For example, Elk-1, ribosomal S6 kinase (RSK)-1 and c-Fos have the following *K*_d_ values for association with ERK2 (values for ppERK2 shown in brackets): 250 nm (> 10 μm), 150 nm (150 nm) and 1 μm (1 μm), respectively [60]. The change in affinity of Elk-1 association when ERK2 is phosphorylated reflects the ability of some MAPK partners to differentiate between nonphosphorylated and phosphorylated forms of the MAPK, which can be important in coordinating function. In this case, it is likely that Elk-1 may recruit ERK2 prior to activation for more efficient phosphotransfer. Although a role for MAPKs in the phosphorylation and activation of transcription factors, such as Elk-1, is well established [61,62], a growing body of evidence suggests that MAPKs are themselves integral components of the transcriptional machinery [63,64], and several roles for MAPKs have been described in the nucleus that do not require kinase activity [65,66]. Unveiling the role of compartment-restricted MKPs in regulating the formation or dissolution of such arrangements remains a major challenge in the MAPK field, but it appears logical to suggest that MKPs may be key regulators in this regard.

A further intriguing feature of MKPs is that, although binding affinity for MAPK substrates is broadly indicative of substrate preference, there are exceptions. For example, DUSP4/MKP-2, which displays a substrate preference for ERK and JNK, actually binds ERK and p38 with higher affinity than JNK [31]. This, in conjunction with experiments comparing relative affinity for MKP–MAPK and MAPK–substrate interactions, raises two important points. First, that the concentration of MKPs relative to substrates is a major determinant of MAPK regulation. Second, that MKPs can either release or anchor substrate MAPKs after dephosphorylation. This points to the MKPs as versatile regulators of both MAPK signal strength and subcellular localization.

## MKP targeting of MAPK: lessons from ERK

Some clear examples of how MKPs may cooperate to regulate MAPK signalling and localization have recently been published in studies of the ERK pathway. Overexpressed, epitope-tagged variants of the nuclear MKPs, DUSP2 (PAC1), DUSP4/MKP-2 and DUSP5, readily coimmunoprecipitate with and cause nuclear accumulation of ERK in the dephosphorylated form, whereas DUSP1/MKP-1 cannot [37,67,68]. DUSP6/MKP-3 also plays an analogous role to the ERK-activating kinase, MEK, by anchoring dephosphorylated ERK in the cytoplasm [50,69,70]. Experiments using Arg–Ala mutants in the MKP KIM, or reciprocal mutations of Asp–Asn in the CD of ERK2, show that the KIM–CD interaction is crucial for these anchoring roles in addition to mediating catalytic activation of the phosphatase [37,50,67]. By contrast, point mutants in the CD do not abrogate MEK-mediated cytoplasmic retention of ERK or MEK-directed phosphorylation, suggesting that MEK binds to ERK in a multivalent manner distinct from that of the MKPs [71–73]. Current models of how DUSP5, DUSP6/MKP-3 and MEK may cooperate to regulate nucleo-cytoplasmic shuttling of ERK are summarized in Fig. 2.

It has been known for some time that sustained (but not transient) activation of MEK often causes nuclear accumulation of ERK in the dephosphorylated form and is coincident with ERK-dependent fate choices such as G_1_/S transition [74]. Inhibitor experiments initially identified that newly synthesized, vanadate-sensitive phosphatases and continuous MEK activity are important for maintaining ERK nuclear localization [74,75]. Transient ERK activation profiles and nuclear localization appear largely uninfluenced by the addition of protein synthesis inhibitors, indicating that this is a feedback control mechanism peculiar to sustained MEK signals [74,76]. This would logically point to the nuclear MKPs as mediators of ERK subcellular targeting during sustained signalling. Loss-of-function experiments using siRNA knockdown of MKPs showed that DUSP2 (PAC1), DUSP4/MKP-2 and DUSP5 are indeed employed in a sustained MEK stimulus-specific manner and regulate nuclear dephosphorylation and nuclear accumulation of ERK [67,76]. A recent study also reported that DUSP4/MKP-2 and DUSP5 are induced in response to oncogenic activation of the ERK pathway in colon cancer-derived cells, which correlates with nuclear accumulation of dephosphorylated ERK. In the absence of commercially available DUSP5 antibodies, the authors further studied DUSP4/MKP-2 in detail, revealing it as a potential regulator of ERK-driven tumour cell proliferation [68]. However, in the absence of knockdown experiments and/or a comparison with DUSP5 (which has much greater substrate selectivity for ERK), it is unclear what unique contribution the two phosphatases make to ERK regulation in the nucleus. The exact function of this nuclear regulation of ERK by MKPs currently unknown, raising questions of whether nuclear-targeted MKP activity is necessary to negotiate G_1_/S transition or participate in oncogenesis. Further questions also remain over whether phosphatases induced by ERK with wider substrate specificity, such as DUSP4/MKP-2, play an important role by dephosphorylating JNK/p38 kinases. We discuss these ideas further below.

## Functions of spatial MKP restriction and crosstalk

There are several reasons why MAPK anchoring by MKPs may have arisen as a control mechanism. Anchored and dephosphorylated MAPKs may be concentrated in subcellular compartments to participate in noncatalytic functions [65,66]. Another explanation is that MKPs could function as a kind of ‘capacitor’, where high concentrations of inactive MAPK are accumulated in regions where they need to be rapidly liberated and reactivated. There are two main mechanisms through which this may be achieved. First, competition for MAPK CD motif association, which would disrupt the MAPK–MKP interaction and allow MKK-mediated reactivation (this may explain how overexpression of the nuclear ERK partner, Mxi2, prolongs ERK phosphorylation responses [77]). Second, rapid alteration of MKP affinity for MAPKs and/or MKP expression [15,16]. There are numerous examples of the latter mechanism, in which different signals rapidly affect MKP turnover and post-translational modification to exert dynamic control over MAPK substrate targeting and crosstalk. Some of the clearest examples of this are summarized below.

Expression of the ERK-specific phosphatases, DUSP5 and DUSP6/MKP-3 in response to growth factor and mitogen stimulation is mediated by ERK activity [78,79]. Because of the intrinsic delay in gene expression, this forms an autoregulatory negative feedback loop in the ERK pathway that is temporally distinct from the more immediate mechanisms of receptor desensitization and ERK-dependent inhibition of upstream regulators that rapidly down-regulate ERK activity [80] (similar to the cartoon in Fig. 3A). Many MKPs are characteristically expressed at low levels under basal conditions and are rapidly induced by stimuli as either immediate early or delayed early genes [81,82]. As such, these MKPs are only likely to influence sustained phases of MAPK responses, whereas MKPs that are constitutively expressed can potentially influence responses at all phases of stimulus. A logical consequence of this type of regulation is that stimuli can cause the cell to have a temporary ‘memory’ through changes in the MKP repertoire, which can desensitize or modulate the effects of subsequent signals [83,84]. This may underpin why transient signals that are limited by rapid mechanisms, such as receptor desensitization and internalization, still induce substantial levels of MKP expression (see Fig. 3B).

Recent evidence derived using genetic and pharmacological tools has highlighted that MKP expression is not simply regulated by MAPK pathways, and has expanded the role of MKPs in signalling crosstalk. Experiments in lymphocytes and hepatocytes showed that transforming growth factor-β induces the expression of DUSP4/MKP-2 through a SMAD3-dependent mechanism. This suppresses ERK-dependent degradation of BIM (Bcl-2-interacting mediator of cell death) and promotes apoptosis [85]. An additional role for SMAD-dependent MKP expression has been identified in the control of mouse embryonic stem cell differentiation. Bone morphogenic protein-4, a member of the transforming growth factor-β superfamily, causes SMAD1/5 and SMAD4-dependent transcription of DUSP9/MKP-4 and dephosphorylation of ERK1/2. This counteracts leukaemia inhibitory factor-induced ERK activation and allows cells to maintain self-renewal in the presence of bone morphogenic protein-4 and leukaemia inhibitory factor [86]. Negative regulators of kinase signalling are critical for maintaining cells close to fate decision boundaries between proliferation and differentiation commitment in order to maximize the resultant number of differentiated cells [87]. The role of MKPs in buffering the competing effects of different cytokines and growth factors on stem cell fate promises to be an intriguing area of study in this regard.

DUSP1/MKP-1 expression can be induced by distinct mechanisms dependent on ERK and/or p38 MAPK activity [44,88–90]. It is likely that both mechanisms give rise to pathway crosstalk because of the substrate preference of DUSP1/MKP-1 for JNK [22]. The biological function of the DUSP1/MKP1 induction by p38 in response to UV/stress responses of fibroblasts is clear in that the apoptotic responses mediated specifically by JNK are curbed [44] in a manner similar to the illustration in Fig. 3C. By contrast, brain-derived neurotrophic factor causes DUSP1/MKP1 expression in cortical neurons in an ERK-dependent fashion during development, which facilitates repression of JNK signalling and axonal branching [91]. Mitogenic ERK-dependent induction of DUSP1/MKP-1 has also been observed in several cell types [74,88]. Although the biological function of DUSP1/MKP-1 in this context is unclear, it is likely that DUSP1/MKP-1 promotes cell survival by suppressing p38/JNK signalling. A similar mechanism appears to be at play during dexamethasone-induced expression of DUSP1/MKP-1 [92]. This response is thought to mediate, in part, the anti-inflammatory effects of glucocorticoids through suppression of p38 activation in macrophages [93,94], but *in vitro* studies indicate that this may additionally cause increased chemoresistance in cancers [95,96]. This correlates with evidence implicating MKP overexpression in cancer progression and drug resistance. The picture here is confusing, with some reports showing that loss or suppression of MKP expression mediates cancer progression, which is more in line with their role as negative regulators of MAPK signalling and intuitive tumour suppressors [97]. Differences here are likely to be dependent upon cancer type and the nature of the driving oncogene, which in turn may cause particular rewiring of MAPK pathways (see below).

## Post-translational regulation of MKPs

Transcriptional regulation is partnered by control of MKP degradation and MAPK affinity by a number of post-translational mechanisms, allowing dynamic coordination of MAPK responses. The ERK-mediated phosphorylation of C-terminal residues (Ser359 and Ser364 in DUSP1/MKP-1) in both DUSP1/MKP-1 and DUSP4/MKP-2 has been shown to result in increased protein stability, thus resulting in positive reinforcement of phosphatase activity and autoregulatory negative feedback control [68,98]. By contrast, ERK-mediated phosphorylation of distinct residues (Ser296 and Ser323) within DUSP1/MKP-1 seems to result in the recruitment of the ubiquitin ligase SCF^skp2^ and increases the rate at which DUSP1/MKP-1 is degraded [99,100]. ERK/mTOR phosphorylation of DUSP6/MKP-3 on Ser159 and ERK phosphorylation of Ser197 residues causes a threefold reduction in DUSP6/MKP-3 half-life, forming a positive feedback loop following mitogenic stimulation [101,102]. Intriguingly, DUSP6/MKP-3 is also a target for pro-apoptotic signalling. Activated caspase 3 cleaves DUSP6/MKP-3 in the conserved central linker region between the NES and catalytic domain, rendering fragments that differentially influence ERK activity and localization. Notably, an N-terminal fragment containing the KIM and NES, but lacking the catalytic domain, competes with full-length DUSP6/MKP-3 for ERK association and increases phosphorylation levels, suggesting a novel mechanism of MKP control during apoptosis [103]. DUSP5 also undergoes ERK-dependent phosphorylation on Thr321, Ser346 and Ser376; the effect of this modification is unclear, but DUSP5 is stabilized by association with ERK substrate in an apparent reinforcement of autoregulation [79]. Clearly, the regulation of MKP stability by phosphorylation is complex and requires further study. However, it is notable that the ERK-dependent phosphorylation sites on DUSP1/MKP-1, DUSP4/MKP-2 and DUSP5 are at the C-terminus, close to the catalytic site of ERK when associated via the N-terminal KIM. For DUSP5, phosphorylation at these sites has been shown to depend upon ERK binding to the KIM [79]. By contrast, it is difficult to see from current structural models of ERK–DUSP6/MKP-3 association via the KIM how Ser159 and Ser197 of DUSP6/MKP-3 would be accessible to the ERK catalytic site. Because the experiments identifying these sites were performed *in vitro* with no possibility of intermediate ERK-dependent kinases fulfilling this role [101], this raises the possibility that additional binding modes between ERK and DUSP6/MKP-3 may occur.

Interestingly, the unique C-terminal stretch of DUSP16/MKP7, a JNK/p38-selective phosphatase [104], is also a target for ERK phosphorylation on Ser446 [105]. This increases the half-life of DUSP16/MKP-7 without influencing its catalytic specificity, causing ERK activity to ultimately suppress JNK signalling [106]. Overexpression experiments have also shown that DUSP16/MKP-7 causes cytoplasmic retention of its chief substrates, JNK and p38 [52], but can surprisingly also serve as an anchor for both phosphorylated and nonphosphorylated forms of ERK and prevent ERK-dependent gene transcription [107]. However, the biochemical basis for the ERK interaction is unresolved, and knockdown of DUSP16/MKP-7 in other systems does not produce effects on ERK localization consistent with this observation (possibly because of the plethora of other ERK scaffolds in the cytosol), leaving the physiological relevance of this mechanism currently unclear [54].

Post-translational changes in and around the N-terminal KIM can also rapidly alter substrate targeting by changing the affinity of the MKP–MAPK interaction. An interesting example of this is the p300-mediated acetylation of the Lys57 residue of DUSP1/MKP-1, which occurs in response to inflammatory toll-like receptor-4 signalling [108]. This modification is immediately adjacent to the critical Arg residues of the KIM, and has the effect of enhancing targeting of MAPK substrates (particularly p38), thus maximizing feedback regulation. The biochemical basis for this enhancement is unclear, but it is likely that acetylation increases the affinity of the N-terminal interaction with target MAPKs, and may increase selectivity for p38 by changing the charge profile around the KIM domain. Recent evidence indicates that this may be an important general mechanism for controlling MKP targeting that is exploited by intracellular pathogens. The enhanced intracellular survival (Eis) protein secreted by *Mycobacterium tuberculosis* (*Mtb*) is able to specifically acetylate the Lys55 residue on DUSP16/MKP-7 (which is analogous to Lys57 on DUSP1/MKP-1) [109]. The authors suggest that the acetyl-transferase activity of Eis causes an enhancement of JNK targeting by DUSP16/MKP-7, thus reducing phosphorylation levels and associated immune functions, which provides a potential explanation for how Eis enhances the intracellular survival of *Mtb* following engulfment by macrophages.

In an analogous mechanism to KIM acetylation, the cytoplasmic ERK and p38 phosphatase, DUSP9/MKP-4, is phosphorylated by protein kinase A on Ser58 [110]. Ser58 is immediately adjacent to the Arg residues that are critical for KIM domain function and phosphorylation of this site most likely abrogates the usual electrostatic interactions occurring at this motif, and prevents the targeting of ERK or p38 substrates by DUSP9/MKP4 [110]. Compared with regulation by effects on MKP turnover, these mechanisms involving modulation of targeting motifs are very rapid and represent key points of integration. DUSP1/MKP-1 acetylation appears to have a drastic effect on innate immune function [111], but the physiological effects of DUSP9/MKP-4 phosphorylation are not yet clear. However, DUSP9/MKP-4 is essential for placental development [112] and has been linked to obesity, insulin resistance and type 2 diabetes in both murine models and human GWAS studies [113–115], highlighting the study of DUSP9/MKP-4 modification in relevant disease models as a key area of investigation.

## Are MKPs tumour suppressors or oncogenes?

A key conundrum is presented by observations of MKP behaviour in cancer progression. As negative regulators of MAPKs, the MKPs represent logical tumour suppressors, and numerous studies correlate loss of MKP expression with progression of several tumour types. However, gain of MKP expression is often associated with cancer progression, drug resistance and poor prognosis [97]. MKP expression may simply be a consequence of elevated MAPK activity, representing a cohort of MAPK-responsive genes that become deregulated in cancer, and which fail to restrain the oncogenic activity of MAPKs, but this would seem unusual given the selection pressure exerted on tumour cells to retain expression profiles conferring fitness [116–118]. Elevated MKPs can block the pro-apoptotic effects of chemotherapeutics, which often act primarily through JNK/p38 stress-activated MAPK pathways [119–122]. Conversely, recent studies show that levels of DUSP4/MKP-2 correlate with drug resistance of residual disease in breast cancer patients following neo-adjuvant chemotherapy. Low levels of DUSP4/MKP-2 expression correlated with higher post neo-adjuvant chemotherapy cancer cell proliferation and a reduction in recurrence-free survival. *In vitro* experiments suggest that this is due to increased ERK activation, because expression of DUSP4/MKP-2 in breast cancer cell lines both reduced ERK activation and increased cell killing by docetaxel. A link to ERK activation is further supported by the observation that the effects of DUSP4/MKP-2 expression can be mimicked by inhibition of MEK [123]. However, some effects of MKPs in cancer may be more subtle and complex. An interesting scenario is presented by studies of cancers driven by the V600E oncogenic mutation of BRAF (B isoform of rapidly accelerated fibrosarcoma kinase). This mutation locks the kinase in a constitutively active form [124], but also blocks a normal inhibitory feedback loop from ERK to BRAF [125–127]. This should drive unrestrained signalling through ERK, but V600E BRAF tumour cells do not have higher levels of ERK activity than cells from other ERK-dependent tumour types. A likely explanation for this is that V600E-driven tumour cells also typically have higher levels of MKPs, which could to some extent compensate for the loss of ERK to BRAF feedback [125,128]. The purpose of this rewiring is currently unclear, but may reflect a necessity for ERK signals to be tempered for them to be fully oncogenic [129,130]. This is consistent with data indicating that high levels of ERK activation causes cell-cycle arrest and senescence [131,132], and that oncogene-induced senescence is potently induced by V600E BRAF [129,130]. The logical implication here is that elevated expression of MKPs in these scenarios enables them to act as tumour promoters rather than suppressors in a manner dependent on the upstream oncogenic mutation, the MKP concentration and the status of other key signalling nodes.

## Summary

It is clear from this update on MKP function that one of the main unanswered questions is a detailed mechanistic understanding of how MKPs maintain spatial control of MAPK signalling. MKP control of MAPK localization and activity in subcellular compartments depends on the rate of MAPK shuttling to and from those compartments, the local concentration of MKP in relation to substrate MAPKs and the on/off rate of the MAPK–MKP interaction. It is also likely that complex interplay exists between the delayed transcriptional feedback exerted by MKPs, such as the action of DUSP4/MKP-2 and DUSP5 on ERK in the nucleus, and other post-translational feedback loops that govern homeostasis, such as ERK phosphorylation of RAF in the cytoplasm [133], and remains a key area for future study. Unravelling the mechanisms by which MKPs maintain strict spatial and temporal control of MAPK activity will require more sophisticated model systems to study the extent to which temporal, global or localized MKP expression is important for shaping MAPK responses and cellular outcomes. However, it is clear that the unique biochemical properties of MKPs puts them centre stage as coordinators of MAPK signalling and crosstalk, as opposed to simple feedback regulators. An additional future challenge is to elucidate how these different mechanisms of MKP regulation influence substrate targeting to integrate the plethora of information that is processed through MAPK cascades. Understanding this will help us to realize how MAPK networks are rewired in disease. While development of specific catalytic inhibitors of MKPs has proven difficult, current efforts to target MAPK docking domains will undoubtedly reveal compounds that specifically inhibit MKPs by inhibiting the CD–KIM interaction [134], promising a new era of MKP therapeutics.

## Abbreviations

BIM: Bcl-2-interacting mediator of cell death
BRAF: B isoform of rapidly accelerated fibrosarcoma kinase
CD: common docking site
CRM-1: chromosome region maintenance-1
DUSP: dual-specificity protein phosphatase
Eis: enhanced intracellular survival
ERK: extracellular signal-regulated kinase
FOXO1: forkhead box O1
JNK: c-jun N-terminal kinase
KIM: kinase interaction motif
MAPK: mitogen-activated protein kinase
MKK: MAPK kinase
MKP: MAP kinase phosphatase
NES: nuclear export signal
NLS: nuclear localization signal
RSK-1: ribosomal S6 kinase -1
STAT: signal transducers and activators of transcription
